# Association of circulating adipsin with nonalcoholic fatty liver disease in obese adults: a cross-sectional study

**DOI:** 10.1186/s12876-021-01721-9

**Published:** 2021-03-21

**Authors:** Jinhua Zhang, Kangli Li, Lingling Pan, Fei Teng, Peizhen Zhang, Bingquan Lin, Youwen Yuan, Xueyun Wei, Wenyuan Li, Huijie Zhang

**Affiliations:** 1 Key Laboratory of Functional and Clinical Translational Medicine, Department of General Medicine, Xiamen Medical College, Xiamen, China; 2 Department of Endocrinology and Metabolism, Nanfang Hospital, Southern Medical University, 1838 North Guangzhou Road, Guangzhou, 510515 China; 3 Department of Endocrinology and Metabolism, Tongji Hospital, Tongji University, Shanghai, China; 4 The First Affiliated Hospital of Xiamen University, Xiamen, China; 5 Department of Medical Imaging Center, Nanfang Hospital, Southern Medical University, Guangzhou, China

**Keywords:** Adipsin, Non-alcoholic fatty liver disease, Obesity, Metabolic syndrome

## Abstract

**Background:**

As a secreted adipokine, adipsin has been recently shown to play a pivotal role in metabolic disorders. However, information regarding the association of circulating adipsin with non-alcoholic fatty liver disease (NAFLD) in humans is scant.

**Methods:**

We recruited 1163 obese adult subjects with waist circumference at least 90 cm in men and 80 cm in women from the community. Circulating adipsin levels were measured by enzyme-linked immunosorbent assay.

**Results:**

Circulating adipsin levels of NAFLD subjects was decreased compared to those in non-NAFLD (*p* < 0.05). The prevalence of NAFLD with lower levels of serum adipsin was significantly higher than those with higher values (57.6% vs. 50.9%, *p* < 0.05). Circulating adipsin levels were significantly associated with decreasing levels of fasting glucose and postprandial glucose (both *p* < 0.001 for interaction) in NAFLD subjects but not in non-NAFLD subjects. The risk of NAFLD was significantly decreased by 21.7% [OR (95% CI): 0.783 (0.679–0.902), *p* < 0.001], adjusting for age, gender, current smoking, alcohol consumption, physical activity, BMI, systolic BP, fasting glucose, total cholesterol, HDL-c, HOMA-IR, and body fat mass. Importantly, subjects in the lowest quartile of circulating adipsin were 1.88 times more likely to have NAFLD than those in the highest quartile in multivariable logistic regression analyses. However, such associations with circulating adipsin were not noted for metabolic syndrome, abnormal liver enzyme and significant liver fibrosis.

**Conclusions:**

These results demonstrate that circulating adipsin levels in Chinese obese adults are negatively associated with risk of NAFLD, implying that serum adipsin levels may be a potential protective factor in NAFLD.

## Introduction

With the epidemic of obesity and effective control of viral hepatitis, non-alcoholic fatty liver disease (NAFLD) has become the most common chronic liver disease in the world [1]. Currently, it estimates approximate 20–30% of adults in Western Countries and 25% in Asia [2]. There is sufficient evidence that obesity and insulin resistance are closely associated with the development of NAFLD in the general population [3]. NAFLD has been recognized as an important risk factor for many related diseases such as liver fibrosis, cirrhosis, type 2 diabetes mellitus (T2DM), cardiovascular (CVD) and cardiac diseases [4].

Numerous studies have shown that adipose tissue, as an endocrine organ, plays a key role in metabolic homeostasis [5]. Adipokines are polypeptides with biological activities secreted by adipose. Some of adipokines are traditional hormones, while others are inflammatory and immune-related cytokines. These cytokines are molecular link between obesity and a variety of pathologies [6]. Previous studies have indicated that several adipokines including leptin, visfatin, fetuin-A, and adiponectin are strongly associated with the development of obesity, type 2 diabetes, metabolic syndrome (MetS) and NAFLD [7–10].

It has been proven that adipsin as a secreted adipokine plays an important role in preserving beta cells through controlling the complement pathway and generation of complement component C3a in diabetic mice, and associates with protection from type 2 diabetes in humans [11]. However, there is little conclusive evidence for association between circulating adipsin and NAFLD in the community cohort study. To our knowledge, only three small case–control studies reported inconsistent findings of associations of circulating adipsin with risk of NAFLD in humans [12–14]. This study aimed to investigate the relationship between circulating adipsin concentrations and the risk of NAFLD in Chinese obese adults.

## Methods

### Study participants

This physical screening program started from 2011 to 2013 and recruited people over 40 years old from the community in China [15]. Those who waist circumference exceeding the standard (90 cm for men or 80 cm for women) have completed a standard questionnaire and a physical examination. Of those, 49 subjects who had consumed more than of 210 g of ethanol (20 alcoholic drinks) per week in men and 140 g of ethanol (10 drinks) in women were excluded. Finally, a total of 1163 adult obese subjects who received an ultrasound were included in the present analysis. Patients who had cancer, currently using systemic corticosteroid therapy, biliary obstructive disease, acute or chronic viral hepatitis, drug-induced liver disease, total parenteral nutrition, autoimmune hepatitis, Wilson's disease or known thyroid function hyperthyroidism or hypothyroidism, would be excluded.

### Diagnosis of fatty liver by ultrasonography and liver fibrosis

All participants had liver ultrasound scans performed by an experienced radiologist who was blinded to the subject's specific medical information/health status. In short, the diagnosis of liver steatosis is based on ultrasound features, including liver and kidney echo contrast, liver parenchymal brightness, deep beam attenuation and blood vessel blurring [16, 17]. However, the semi-quantitative sonographic scoring for the degree of hepatic steatosis was not included in this study. According to guidelines, fibrosis-4 (FIB-4) index was used to determine liver fibrosis severity, which also could be used in patients with normal transaminases [18–21]. The FIB-4 index was calculated using following formular: age (years) × AST (U/L)/(platelet count [×10^9^/L] × ALT [U/L]^1/2^). Participants was classified into low-risk (FIB index < 1.30), intermediate-risk (FIB index: 1.30–2.67) and high-risk (FIB index > 2.67) groups for advanced fibrosis [18]. We defined who had progressed to significant liver fibrosis by FIB-4 index ≥ 1.30 according to previous study [22].

### Clinical and biochemical measurements

The details of the clinical and biochemical examinations of the included subjects in the study have been reported in previous reports [23]. Subjects’ weight and height were measured by spring scale and vertical ruler, respectively. Body mass index (BMI) was calculated by dividing the weight in kilograms by the square of the height in meters. Waist circumference was measured in triplicate at the level of the umbilicus with a nonstretchable tape in the morning. Body fat mass was assessed by DXA (Hologic Inc., Bedford, MA).

All subjects were instructed to fast for 12 h before screening. As previously described [23] Blood biochemical measurements were performed on each subject, including a 75 g oral glucose tolerance test. Serum triglycerides (TG), total cholesterol (TC) and high-density lipoprotein cholesterol (HDL-c) were measured by using an enzyme colorimetric method performed on an automatic multi-channel chemical analyzer (Hitachi 7450, Tokyo, Japan). Use Friedwald's formula to calculate serum low-density lipoprotein cholesterol (LDL-c). Serum alanine aminotransferase (ALT) and aspartate aminotransferase (AST) were quantified using standard enzymatic methods. The Szasz-Persijn method was used to quantify serum γ-glutamyltransferase (GGT). Fasting serum insulin (µU/mL) × fasting plasma glucose (mmol/L)/22.5 is used to evaluate the insulin resistance homeostasis model evaluation (HOMA-IR) to evaluate the insulin resistance status.

### Circulating Adipsin measurement

Enzyme‐linked immunosorbent assay kits (AssayPro, St. Charles, MO, USA) were used to detected circulating adipsin. The standard linear range was 0.001875–0.120 ug/mL, and variations were less than 10%.

### Statistical analysis

Continuous variables were summarized using means ± standard deviation (S.D) or median (interquartile range). Categorical variables were presented as number and percentage. Metabolic syndrome (MetS) was defined by the criteria of International diabetes Federation (IDF) [24], Data that were not normally distributed were logarithmically transformed before analysis. For the analysis of categorical variables, the differences in different study groups were compared by Chi-square test or logistic regression models. General linear models (GLM) were performed to test differences in study variables between NAFLD and non-NAFLD groups, and quartiles of circulating adipsin. Linear regression analyses were performed to determine the correlation of circulating adipsin levels with metabolic risk factors. Abnormal ALT, AST and GGT were defined as values exceeding the upper normal level. The association of circulating fat content with the risk of MetS, NAFLD, abnormal liver function, and severe liver fibrosis was examined using a multivariate logistic regression model. The modification effect of presence of NAFLD on the association between circulating adipsin and plasma glucose levels was examined by multivariable linear regression interaction models using the entire sample. *p *values < 0.05 were considered statistically significant. Analyses were performed by using SAS 9.4 (SAS Institute, Cary, NC).

## Results

**Fig. 1:**
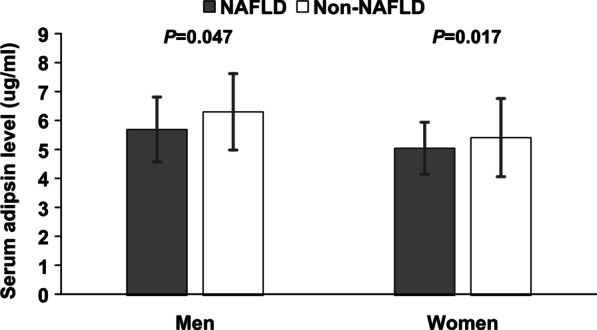
Serum adipsin levels in NAFLD and Non-NAFLD subjects

Table 1 summarized the basic characteristics of study participants. Higher BMI, waist circumference, fasting plasma glucose, postprandial glucose, BP, TG, total cholesterol, liver enzymes, body fat mass, and HOMA-IR levels were found in NAFLD than those in controls. Moreover, NAFLD subjects had lower levels of HDL-c. It is worth noting that the circulating adipsin level of NAFLD subjects was lower than that of non-NAFLD subjects [5.24 ± 1.97 ug/mL vs. 5.57 ± 2.70 ug/mL, *p* < 0.05]. As shown in Fig. 1, There were similarities in circulating adipsin levels between NAFLD and non-NAFLD subjects (*p* > 0.05).

**Table 1:** Clinical characteristics of obese subjects by non-alcoholic fatty liver disease (NAFLD)

Variables	Overall	NAFLD	Non-NAFLD	*p* value
Sample size	1163	657	506	
Age (years)	53.2 ± 7.3	53.7 ± 7.3	52.6 ± 7.2	*0.008*
Gender (male n, %)	299 (25.7)	207 (34.0)	92 (21.9)	*<* *0.001*
BMI (kg/m^2^)	27.4 ± 3.0	28.2 ± 3.1	26.4 ± 2.5	*<* *0.001*
Waist circumference (cm)	93.4 ± 7.0	95.3 ± 7.2	91.1 ± 5.9	*<* *0.001*
Current smokers (n, %)	135(12.6)	91(14.5)	44(10.2)	*0.006*
Systolic BP (mmHg)	133.3 ± 17.8	136.3 ± 17.7	129.4 ± 17.3	*<* *0.001*
Diastolic BP (mmHg)	79.2 ± 10.6	81.1 ± 10.6	76.7 ± 10.1	*<* *0.001*
Triglycerides (mmol/L)	1.53 (1.03–2.22)	1.84 (1.32–2.57)	1.18 (0.86–1.71)	*<* *0.001*
Total cholesterol (mmol/L)	5.89 ± 1.07	6.00 ± 1.07	5.74 ± 1.05	*<* *0.001*
LDL- cholesterol (mmol/L)	3.68 ± 0.97	3.72 ± 1.01	3.64 ± 0.92	0.161
HDL-cholesterol (mmol/L)	1.37 ± 0.30	1.31 ± 0.27	1.45 ± 0.31	*<* *0.001*
Fasting glucose (mmol/L)	6.08 ± 1.64	6.28 ± 1.90	5.82 ± 1.19	*<* *0.001*
2-h glucose (mmol/L)	8.81 ± 3.81	9.58 ± 4.13	7.80 ± 3.07	*<* *0.001*
HOMA-IR	2.88 (2.09–4.16)	3.46 (2.49–5.00)	2.38 (1.68–3.24)	*<* *0.001*
ALT (U/L)	24.4 ± 14.7	27.8 ± 17.0	20.1 ± 9.5	*<* *0.001*
AST (U/L)	22.2 ± 7.3	23.4 ± 8.1	20.7 ± 5.9	*<* *0.001*
GGT (U/L)	34.2 ± 22.7	38.8 ± 24.0	28.2 ± 19.4	*<* *0.001*
Serum adipsin (ug/ml)	5.39 ± 2.32	5.24 ± 1.97	5.57 ± 2.70	*0.017*
Body fat mass(kg)	24.1 ± 5.3	25.0 ± 5.6	23.0 ± 4.7	*<* *0.001*
FIB-4 value	0.95 ± 0.41	0.93 ± 0.38	0.97 ± 0.45	*0.158*
Liver fibrosis (n, %)				*0.317*
FIB-4 Low	991 (85.2)	566 (86.2)	425 (84.0)	
FIB-4 Intermediate	167 (14.4)	87 (13.2)	80 (15.8)	
FIB-4 High	5 (0.4)	4 (0.6)	1 (0.2)	

Data are presented as the mean ± SD or median (interquartile range)

BMI = body mass index; HOMA-IR = homeostasis model assessment of insulin resistance; NAFLD = nonalcoholic fatty liver; FIB-4 = fibrosis-4 index

The clinical characteristic by quartiles of circulating adipsin levels were shown in Table 2. After adjustment for age and gender, there were no significant differences in BP, TG, total cholesterol, LDL-C, HDL-C, postprandial blood glucose, ALT, GGT, HOMA-IR, and FIB-4 among the four quartiles of circulating adipsin levels. Subjects in the highest quartile of circulating adipsin levels had significantly higher levels of waist circumference, BMI, AST and body fat mass (all *p* < 0.05) than those in the lowest quartile. Additionally, fasting glucose and postprandial glucose were reduced gradually with the increase of circulating adipsin (*p* < 0.001 and *p* = 0.039, respectively). Interestingly, the prevalence of NAFLD in subjects with lower levels of circulating adipsin was significantly higher than those with higher values (57.6% vs. 50.9%, *p* < 0.05). However, prevalence of MetS did not differ significantly across quartiles of circulating adipsin levels.

**Table 2:** Clinical characteristics by quartiles of serum adipsin levels in obese subjects

Variables	Serum adipsin level				*p* value for trend^§^
	Quartile 1	Quartile 2	Quartile 3	Quartile 4	
Sample size	290	291	291	291	
Serum adipsin (ug/ml)	3.27 ± 0.64	4.45 ± 0.35	5.51 ± 0.46	8.30 ± 2.63	*<* *0.001*
Age (years)	51.9 ± 7.2	52.6 ± 7.2	53.6 ± 7.5^‡^	54.8 ± 7.1^‡^	*<* *.0001*
Gender (male n, %)	74 (25.5)	75 (25.8)	75 (25.8)	75 (25.8)	1.000
BMI (kg/m^2^)	26.9 ± 2.7	27.1 ± 2.6	27.6 ± 3.3^†^	28.0 ± 3.3^‡^	*<* *0.001*
Waist circumference (cm)	92.2 ± 6.2	93.2 ± 6.9	93.8 ± 7.3^†^	94.6 ± 7.4^‡^	*<* *0.001*
Current smokers (n, %)	34 (11.7)	36 (12.4)	35 (12.0)	30 (10.3)	0.822
Systolic BP (mmHg)	132.3 ± 16.9	132.4 ± 17.6	133.9 ± 18.4	134.5 ± 18.3	0.864
Diastolic BP (mmHg)	78.5 ± 10.4	79.3 ± 10.2	79.4 ± 11.1	79.6 ± 10.7	0.599
Triglycerides (mmol/L)	1.42 (0.96–2.19)	1.61 (1.13–2.31)	1.43 (0.99–2.19)	1.61 (1.09–2.20)	0.128
Total cholesterol (mmol/L)	5.88 ± 1.08	5.86 ± 1.01	5.90 ± 1.07	5.92 ± 1.10	0.955
LDL-cholesterol (mmol/L)	3.67 ± 1.01	3.59 ± 0.95	3.73 ± 0.92	3.74 ± 1.01	0.491
HDL-cholesterol(mmol/L)	1.39 ± 0.30	1.36 ± 0.31	1.38 ± 0.28	1.34 ± 0.29	0.182
Fasting glucose (mmol/L)	6.40 ± 2.47	6.05 ± 1.52^‡^	5.98 ± 1.15^‡^	5.89 ± 0.98^‡^	*<* *0.001*
2-h glucose (mmol/L)	9.23 ± 4.74	8.74 ± 3.65	8.69 ± 3.31^†^	8.57 ± 3.34^†^	*0.039*
HOMA-IR	3.09 (2.16–4.46)	2.89 (2.16–4.28)	2.65 (1.97–3.76) ^†^	2.83(2.03–4.14)	*0.035*
ALT (U/L)	24.2 ± 12.2	24.9 ± 13.1^†^	25.8 ± 19.8	22.9 ± 12.3	0.107
AST (U/L)	21.7 ± 7.1	22.0 ± 6.5	23.4 ± 9.1	21.9 ± 6.2	*0.030*
GGT (U/L)	35.1 ± 23.9	35.0 ± 23.4	34.2 ± 23.3	32.3 ± 20.0	0.404
Body fat mass (kg)	23.6 ± 5.1	23.9 ± 5.0	24.2 ± 5.4	24.8 ± 5.6^‡^	*0.003*
FIB-4 value	0.90 ± 0.37	0.94 ± 0.52	0.98 ± 0.37	0.97 ± 0.36	0.608
Metabolic syndrome (n, %)	184 (63.5)	183 (63.1)	181 (62.2)	196 (67.4)	0.579
NAFLD (n, %)	169 (58.3)	175 (60.1)	167 (57.4)	146 (50.2) ^†^	*0.012*

Data are presented as the mean ± SD or median (interquartile range)

BMI = body mass index; HOMA-IR = homeostasis model assessment of insulin resistance; FIB-4 = fibrosis-4 index

^§^Adjusted for age and gender

^†^*p* < 0.05compared with Q1 of serum adipsin

^‡^*p* < 0.01compared with Q1 of serum adipsin

**Fig. 2:**
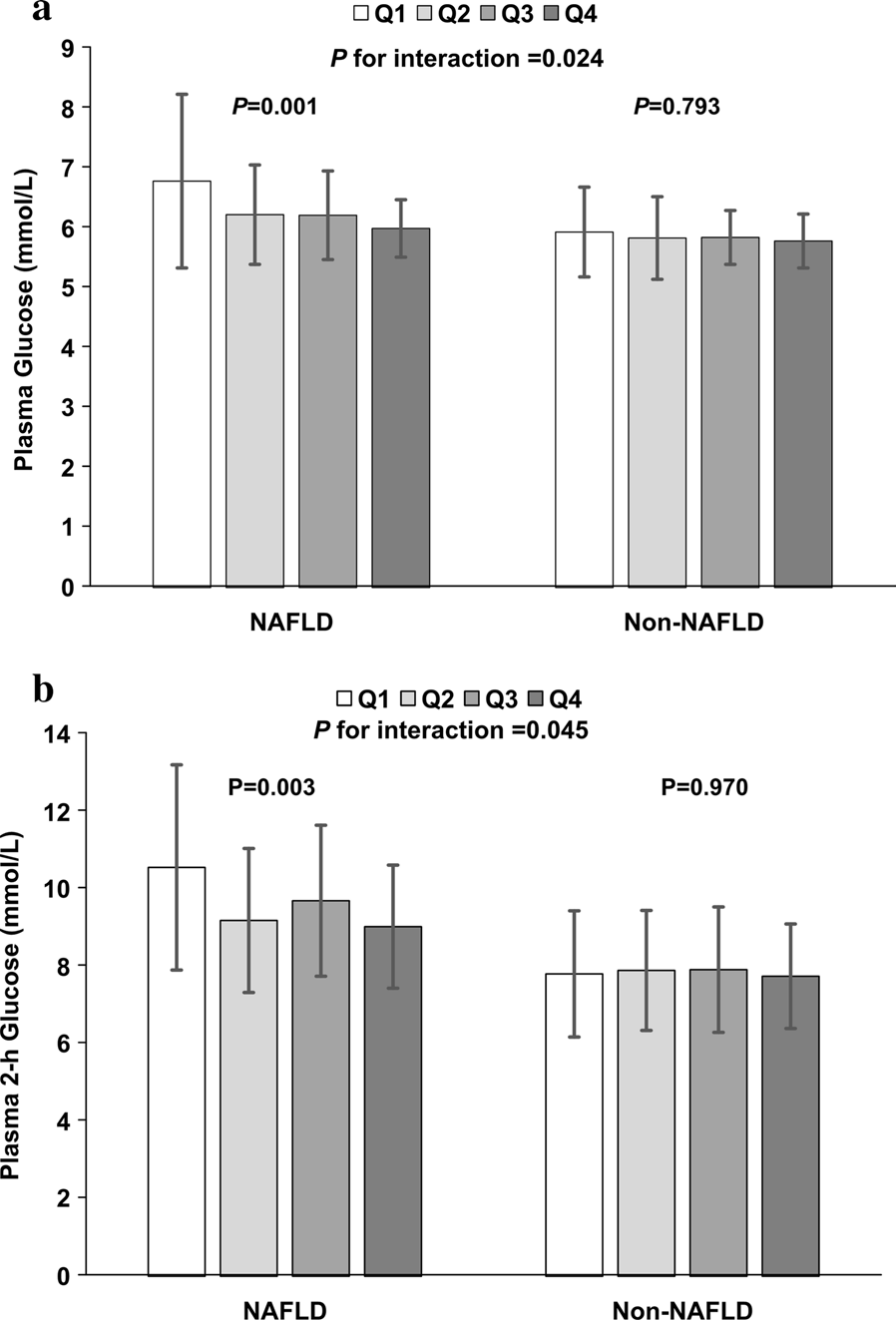
Fasting plasma glucose and plasma 2-h glucose according to quartiles of serum adipsin levels in NAFLD and Non-NAFLD subjects. **a** Fasting plasma glucose according to quartiles of serum adipsin levels. **b** Plasma 2-h glucose according to quartiles of serum adipsin levels

As shown in Fig. 2, fasting blood glucose in NAFLD subjects decreased gradually with the increase of circulating adipsin (*p* = 0.001), however, there was no significant difference in non-NAFLD groups (*p* = 0.793 for non-NAFLD; *p* = 0.024 for interaction). Likewise, postprandial 2-h glucose showed negative association with circulating adipsin levels in NAFLD subjects (*p* = 0.003) but no significant association in the non-NAFLD group (*p* = 0.970 for non-NAFLD; *p* < 0.045 for interaction).

Table 3 shows the relationship of circulating adipsin levels with metabolic risk factors parameters in linear regression analyses. Circulating adipsin was positively correlated with BMI, waist circumference and body fat mass in NAFLD group (all *p* < 0.05), adjusting for age, gender, smoking, alcohol consumption, and physical activity. In addition, circulating adipsin showed negatively association with fasting glucose, postprandial 2-h glucose, GGT, and HOMA-IR in NAFLD group (all *p* < 0.05). Meanwhile, such significant association was not shown in non-NAFLD groups.

**Table 3:** Clinical correlates of serum adipsin levels with NAFLD-associated metabolic risk factors and liver fibrosis score

Variables	Total			NAFLD			Non-NAFLD		
	β	*p* value	Multiple adjusted *p* value^§^	β	*p* value	Multiple adjusted *p* value^§^	β	*p* value	Multiple adjusted *p* value^§^
Age(years)	1.192	*<* *0.001*	–	1.430	*<* *0.001*	–	1.033	*<* *0.001*	–
Gender (male n, %)	–	*<* *0.001*	–	–	*<* *0.001*	–	–	*<* *0.001*	–
BMI (kg/m^2^)	0.352	*<* *0.001*	*<* *0.001*	0.607	*<* *0.001*	*<* *0.001*	0.199	*0.046*	*0.027*
Waist circumference (cm)	1.034	*<* *0.001*	*0.001*	1.557	*<* *0.001*	*<* *0.001*	0.741	*0.002*	*0.045*
Systolic BP (mmHg)	1.550	*0.003*	0.283	1.713	*0.025*	0.417	1.662	*0.016*	0.196
Diastolic BP (mmHg)	0.938	0.003	*0.033*	1.022	*0.026*	0.092	1.028	*0.011*	0.039
Total cholesterol (mmol/L)	0.127	0.686	0.523	− 0.019	0.678	0.309	0.049	0.239	0.625
Total triglycerides(mmol/L)	0.008	0.823	0.425	0.013	0.825	0.748	0.032	0.433	0.792
LDL-cholesterol(mmol/L)	0.035	0.218	0.808	0.007	0.870	0.707	0.062	0.092	0.384
HDL-cholesterol(mmol/L)	− 0.025	*0.003*	0.100	− 0.030	*0.009*	0.055	− 0.026	*0.033*	0.172
Fasting glucose (mmol/L)	− 0.235	*<* *0.001*	*<* *0.001*	− 0.433	*<* *0.001*	*<* *0.001*	− 0.050	0.289	0.064
2 h glucose (mmol/L)	− 0.338	*0.003*	*<* *0.001*	− 0.723	*<* *0.001*	*<* *0.001*	0.056	0.649	0.939
ALT (U/L)	0.114	0.792	0.420	0.310	0.674	0.873	0.227	0.552	0.988
AST (U/L)	0.328	0.127	0.902	0.550	0.114	0.563	0.234	0.323	0.797
GGT (U/L)	− 0.172	0.796	0.060	− 0.621	0.549	*0.072*	0.596	0.444	0.967
HOMA-IR	− 0.159	0.044	*0.024*	− 0.373	*0.002*	*0.006*	0.074	0.446	0.672
Body fat mass(kg)	0.070	0.654	*0.002*	0.090	0.715	*0.003*	0.140	0.459	*0.005*
FIB-4 value	0.036	0.003	0.728	0.050	0.003	0.450	0.023	0.201	0.741

BMI = body mass index; HOMA-IR = homeostasis model assessment of insulin resistance; FIB-4 = fibrosis-4 index

^§^Adjusted for age, gender, smoking, alcohol consumption, and physical activity

Table 4 presents the multivariable-adjusted odds ratios (ORs) for the relationship between circulating adipsin and risks of NAFLD, MetS, abnormal liver function, and significant liver fibrosis. After adjustment for age, gender, current smoking, alcohol consumption, and physical activity, elevated circulating adipsin levels were closely related to reduced risk of NAFLD [OR (95% CI): 0.850(0.752–0.961), *p* = 0.010]; however, circulating adipsin was not significantly associated with risks of elevated serum ALT, AST and GGT, MetS, and significant liver fibrosis. Furthermore, increased circulating adipsin levels were significantly associated with reduced risk of NAFLD [OR (95% CI): 0.766(0.666–0.882), *p* < 0.001], even after adjusting for risk factors for age, gender, current smoking, alcohol consumption, physical activity, BMI, systolic BP, fasting glucose, TG, and HDL-c; nevertheless, such associations of circulating adipsin levels were not noted for risks of elevated serum ALT, AST, GGT and MetS, and significant liver fibrosis. The correlation between circulating adipsin and NAFLD was still significant after further adjustment for HOMA-IR and body fat mass [OR (95% CI): 0.783 (0.679–0.902), *p* < 0.001].

**Table 4:** Odds ratios of abnormal liver function and NAFLD according to serum adipsin

	OR	95% CI	*p* value
*Model 1*			
Elevated serum ALT	0.923	0.751–1.136	0.450
Elevated serum AST	1.047	0.705–1.554	0.821
Elevated serum GGT	0.917	0.775–1.086	0.316
NAFLD	0.850	0.752–0.961	*0.010*
Metabolic syndrome	1.027	0.905–1.165	0.680
Significant liver fibrosis by FIB-4	1.008	0.847–1.200	0.927
*Model 2*			
Elevated serum ALT	0.863	0.687–1.083	0.203
Elevated serum AST	1.005	0.652–1.548	0.984
Elevated serum GGT	0.914	0.756–1.104	0.350
NAFLD	0.766	0.666–0.882	*<* *0.001*
Metabolic syndrome	0.940	0.779–1.134	0.518
Significant liver fibrosis by FIB-4	1.038	0.866–1.244	0.687
*Model 3*			
Elevated serum ALT	0.872	0.691–1.000	0.248
Elevated serum AST	1.016	0.653–1.581	0.944
Elevated serum GGT	0.916	0.757–1.109	0.370
NAFLD	0.783	0.679–0.902	*<* *0.001*
Metabolic syndrome	0.968	0.800–1.170	0.733
Significant liver fibrosis by FIB-4	1.036	0.864–1.243	0.702

Model 1: adjusted for age, gender, smoking, alcohol consumption, and physical activity. Model 2: adjusted for model 1 + BMI, SBP, glucose, total cholesterol, triglyceride, and HDL-c. Model 3: adjusted for model 2 + HOMA-IR and body fat mass

OR = odds ratio; CI = confidence interval; BMI = body mass index; HOMA-IR = homeostasis model assessment of insulin resistance; FIB-4 = fibrosis-4 index

The multivariable-adjusted ORs for increased risk of NAFLD are shown in Table 5. According to quartiles of circulating adipsin levels, subjects in the lowest quartile of circulating adipsin were 1.88 times (*p* < 0.001) more likely to have NAFLD than those in the highest quartile. Similarly, the risk of NAFLD was significantly increased in the second and third quartiles compared to those in the fourth quartile (all *p* < 0.001). However, the association of quartiles of circulating adipsin levels with significant liver fibrosis defined by FIB-4 index was not significant in the multivariate logistic regression models.

**Table 5:** Odds ratios for NAFLD according to quartiles of serum adipsin levels, adjusted for covariates^§^ in logistic regression models

	NAFLD		Significant liver fibrosis	
	OR (95% CI)	Multivariate-adjusted *p* value	OR (95% CI)	Multivariate-adjusted *p* value
Q1 versus Q4	1.88 (1.26–2.81)	–	0.99 (0.63–1.53)	–
Q2 versus Q4	1.88 (1.26–2.79)	–	1.31 (0.86–2.00)	–
Q3 versus Q4	1.70 (1.15–2.50)	–	1.44 (0.95–2.17)	–
(Q1 + Q2 + Q3) versus Q4	1.81 (1.31–2.51)	*<* *0.001*	1.25 (0.88–1.77)	*0.213*

OR = odds ratio; CI = confidence interval; Q = quartile

^§^Adjusted for age, sex, smoking, physical activity, BMI, Systolic BP, glucose, total cholesterol, HDL-c, HOMA-IR, and body fat

## Discussion

Adipsin is recently identified as a novel adipokine that may play a key role in the development of obesity-associated metabolic diseases, including T2DM and MetS [25, 26]. In the current study, we provide for the first evidence that lower circulating adipsin levels were independently associated with increased risk of NAFLD in obese Chinese adults. Importantly, circulating adipsin levels were significantly associated with decreasing levels of fasting glucose and postprandial glucose in NAFLD subjects but not in non-NAFLD subjects. In addition, our data indicated that serum adipsin could be a predictive factor for NAFLD, suggesting that it would be useful to distinguish the diverse obese phenotypes, including metabolically healthy obesity and metabolically abnormal obesity [27]. These findings have important clinical and public health implication for targeted preventive strategies in practice.

Studies have shown that overweight/obese subjects have higher circulating adipsin levels. Adipsin levels were also associated with an increased cardiovascular risk in patients with polycystic ovary syndrome [28, 29]. Our study findings that circulating adipsin positively associated with BMI and waist circumference were consistent with previous study [11]. Additionally, circulating adipsin levels were significantly associated with body fat mass in our study. Similarly, it has been shown that adipsin was associated with subcutaneous but not visceral adiposity [11]. Given that obesity is highly associated with insulin resistance, it may play a key role in the occurrence and development of MetS and NAFLD[26]. A case–control study reported that subjects with MetS had higher circulating adipsin levels than healthy controls [26]. Unfortunately, our study did not find a significant association of circulating adipsin levels with risks of MetS and components of MetS in obese adults, including blood pressure, hyperglycemia, and lipid profiles. Furthermore, Jun-Sing Wang and colleague reported that circulating adipsin levels were negatively associated with insulin resistance in 320 subjects with various degrees of glucose intolerance, especially in subjects with a BMI ≥ 25 kg/m^2^ or prediabetes [30]. Consistently, our findings indicated that circulating adipsin levels were inversely correlated with HOMA-IR in NAFLD subjects but not in non- NAFLD subjects.

Of interest, our study indicated that circulating adipsin levels were significantly decreased in NAFLD subjects compared to non-NAFLD subjects. However, there is little conclusive evidence regarding the associations between circulating adipsin and NAFLD in human. Up to now, only three small case–control studies reported inconsistent findings of associations of circulating adipsin with risk of NAFLD in humans [12–14]. Yilmaz et al. and other study reported that circulating adipsin levels were no significantly different in biopsy-proven NAFLD subjects versus healthy control [12, 13]. In contrast, Qiu Y et al. reported that circulating adipsin levels were significantly associated with increased risks of NAFLD in 100 NAFLD subjects compared to controls [14]. Limited evidence is based on case–control study designs, and all the studies above had small sample sizes (less than 200 subjects). In this study, sample size was over 1,000 obese adults. We found that circulating adipsin levels are inversely associated with risk of NAFLD in obese Chinese adults. Our data demonstrated that each SD increase in circulating adipsin levels was associated with a 27% decrease in the risk of NAFLD. These data indicated that adipsin insufficiency may be a common feature of NAFLD in obese individuals. Furthermore, there was little evidence regarding the associations between circulating adipsin and liver fibrosis. Unfortunately, our study found no significant associations of circulating adipsin levels and FIB-4 index, which reflects the severity of liver fibrosis.

In addition, growing evidence suggests that adipsin preserves beta cells through controlling the complement pathway and generation of complement component C3a in diabetic mice, and associated with protection from type 2 diabetes in humans [11, 25]. A longitudinal cohort study reported that higher adipsin levels were associated with a lower risk of incident diabetes in 5,570 middle-aged adults [11]. Several clinical studies indicated that low levels of circulating adipsin were associated with β cell failure and poor glycemic control in Type 2 diabetes [11, 31]. Consistently, circulating adipsin levels were negatively associated with fasting glucose and postprandial glucose in our study. Importantly, these associations of circulating adipsin levels with fasting and postprandial glucose levels are significant in NAFLD subjects but not in non-NAFLD subjects. These findings suggest that adipsin may be a potential treatment and predictive marker for type 2 diabetes.

There are several putative mechanisms linking circulating adipsin to risk of NAFLD in obese adult. First, high levels of circulating adipsin are significantly associated with insulin-sensitive obese individuals in the present study, which may be protected from increased risks of NAFLD and T2DM [11]. Therefore, it is no surprise that circulating adipsin levels could predict metabolic profiles of obesity and hepatic fat accumulation. Second, adipsin may involve a direct connection between this molecule and hepatic lipid metabolism. In this regard, circulating adipsin may regulate the complement replacement pathway to catalyze the production of the C3a and significantly inhibit expression of gluconeogenic gene, such as Pepck and G6pc [25]. These findings suggest that it is possible that adipsin or its products may have additional direct effects on the liver. Therefore, circulating adipsin may be a predictive factor for at-risk metabolic phenotype in obese subjects. Prospective cohort studies are needed to support this finding and clarify the potential underlying mechanisms.

This community-based study provided an opportunity to determine the role of circulating adipsin in predicting the development of NAFLD and associated metabolic disorders. The current study has several limitations. First, the population in this cross-sectional study consisted of only obese adults and the sample size was relatively limited. It has been known that adipsin is positively correlated excess adiposity and traditional metabolic risk factors, which may confound the association of adipsin and clinical outcomes. However, we show independent associations of circulating adipsin and outcomes even after accounting for multiply potential confounders. Further studies need to investigate the relationships of circulating adipsin with the development of NAFLD in the general population. Second, the causal relationship between circulating adipsin and the development of NAFLD could not be determined due to its cross-section design. Third, in this study, NAFLD was determined by liver ultrasonography scanning instead of Magnetic resonance imaging, transient elastography or liver biopsy. Considering that hepatic steatosis less than 20% would be underdiagnosed as non-NAFLD by liver ultrasonography scanning ([32–34], the difference between NAFLD and non-NAFLD groups might be weaken or underestimated in the current study. Finally, the study was conducted with obese Chinese adults. Therefore, the causal relationship should be confirmed in prospective cohort studies with larger sample sizes and longer follow-up periods in general population studies.

## Conclusions

In summary, these results indicate that circulating adipsin levels are negatively correlated with the risk of NAFLD in Chinese obese adults, suggesting that serum adipsin levels may be a potential protective factor for NAFLD and would be useful to distinguish the diverse obese phenotypes.

## Data Availability

The datasets used and/or analysed during the current study are available from the corresponding author on reasonable request.

## Abbreviations

NAFLD: Non-alcoholic fatty liver disease
T2DM: Type 2 diabetes mellitus
CVD: Cardiovascular Disease
FGF21: Fibroblast growth factor-21
MetS: Metabolic syndrome
BMI: Body mass index
BP: Blood pressure
TG: Triglyceride
TC: Total cholesterol
HDL-c: High-density lipoprotein cholesterol
LDL-c: Low-density lipoprotein cholesterol
ALT: Alanine aminotransferase
AST: Aspartate aminotransferase
GGT: Gamma-glutamyltransferase
HOMA-IR: Homeostasis model assessment of insulin resistance

## Declarations

### Competing of interest

No potential competing of interest were disclosed.
